# Fault-Tolerant Six-DoF Pose Estimation for Tendon-Driven Continuum Mechanisms

**DOI:** 10.3389/frobt.2021.619238

**Published:** 2021-04-30

**Authors:** Antonin Raffin, Bastian Deutschmann, Freek Stulp

**Affiliations:** German Aerospace Center (DLR), Robotics and Mechatronics Center, Weßling, Germany

**Keywords:** pose estimation, fault-tolerant, data-driven, machine learning, continuum mechanism

## Abstract

We propose a fault-tolerant estimation technique for the six-DoF pose of a tendon-driven continuum mechanisms using machine learning. In contrast to previous estimation techniques, no deformation model is required, and the pose prediction is rather performed with polynomial regression. As only a few datapoints are required for the regression, several estimators are trained with structured occlusions of the available sensor information, and clustered into ensembles based on the available sensors. By computing the variance of one ensemble, the uncertainty in the prediction is monitored and, if the variance is above a threshold, sensor loss is detected and handled. Experiments on the humanoid neck of the DLR robot DAVID, demonstrate that the accuracy of the predicted pose is significantly improved, and a reliable prediction can still be performed using only 3 out of 8 sensors.

## 1 Introduction

**FIGURE 1:**
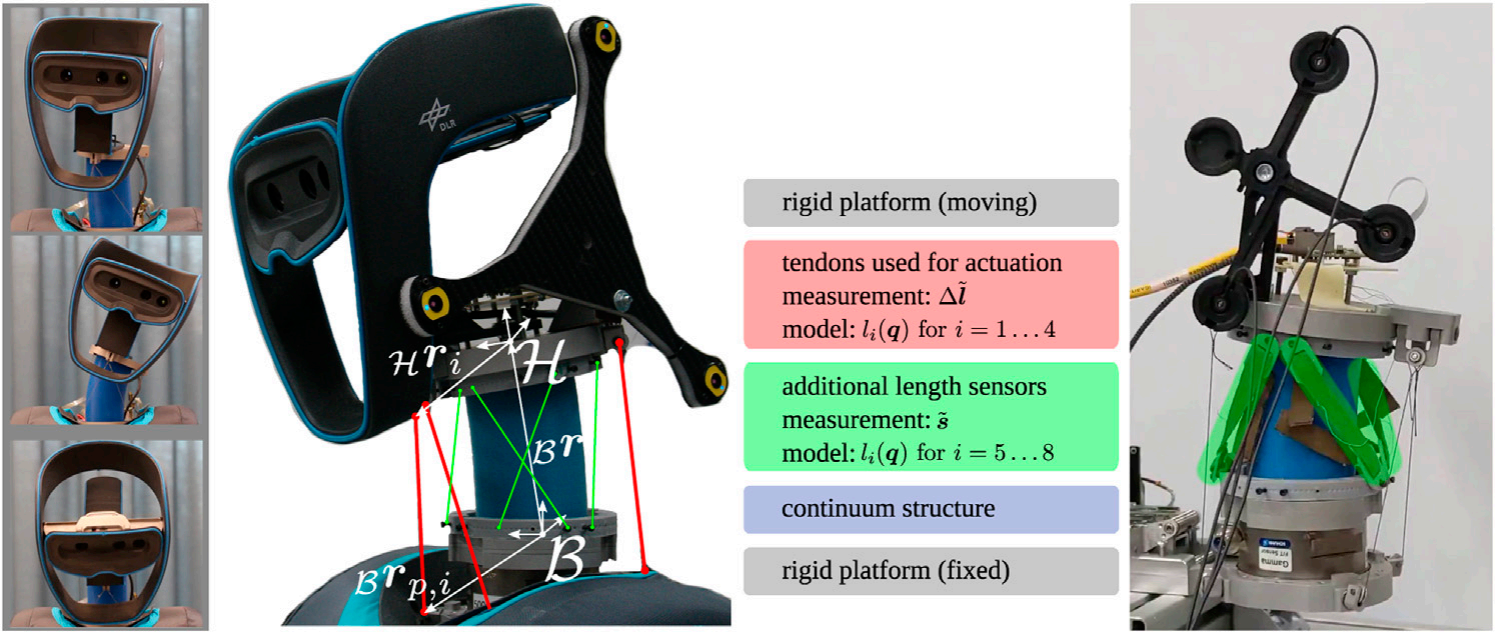
Neck and head of the humanoid robot David. **Left:** Motion capability of the structurally flexible neck joint. **Middle:** Close up scheme of the experimental system and the necessary length information from the respective sensors. **Right:** Experimental setup with the LEDs used by the tracking system (in black) and the additional length sensors (highlighted in green).

Robotic systems with deliberately introduced elasticity have become a promising alternative to classical rigid robots, be it in manipulation or locomotion tasks, in humanoid or animoid robots. Apart from their favorable dynamic properties, passive compliance protects the actuators from peak forces or torques that arise from collisions, be they unexpected or intentional. In this context, the development of distinct joints arose termed continuum mechanism. They extend the current portfolio of joints with passive compliance and use a spring element made out of a continuously deformable soft material with a beam-like geometry. As an example, the neck joint of the humanoid robot David (Reinecke et al., 2016) is shown in Figure 1.

The application requires to control the pose of the top end of the continuum mechanism, termed end effector pose, along trajectories or toward equilibrium positions in its workspace. Rigorous models which relate encoder values of the actuation to end effector poses are computationally expensive and prone to model parameter uncertainties. As we are aiming for mobile systems, the need of external tracking system must be limited to calibration and evaluation of a pose estimation method. At deployment time, such pose estimation method must use onboard sensors only.

In this paper, we propose a data-driven approach to pose estimation for elastic structures, which also features uncertainty estimation and error-handling. The specific contributions, which also define the structure of the paper, are:

• A data-driven approach for real time six-DoF pose of tendon-driven continuum mechanism using few data points. The model-free estimator can be trained in the experiment and requires only a small number of measurements (Section 4.1).
• Uncertainty estimation to detect sensor failure (e.g., slack tendons). This is done by creating an ensemble of estimators, where each estimator takes only a subset of sensor information as an input, and monitoring the uncertainty (Section 4.3).
• A strategy to handle sensor by adapting the pose estimation. As soon as an anomaly is detected, we select the estimators not using the faulty sensors and continue predicting accurately the pose. To the best of our knowledge, this is the first work dealing with failure detection and handling in the context of continuum mechanisms (Section 4.3).
• Demonstrating the effectiveness of these methods on the elastic neck of the DLR robot DAVID. In particular, we demonstrate that the accuracy of the pose estimation is significantly improved, and that reliable predictions can still be made with only 3 out of 8 sensors (Section 5).

Before following this structure, we first formalize the problem statement in the next section.

## 2 Problem Statement

**FIGURE 2:**
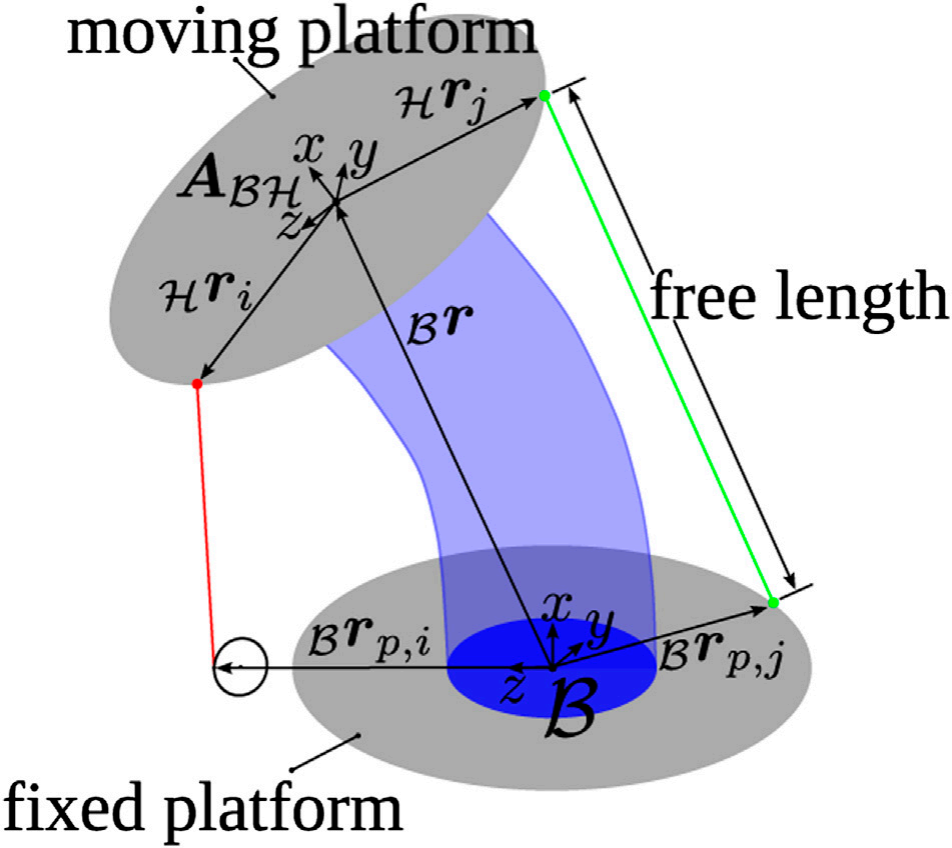
Schematic image of the tendon-driven continuum mechanism including the used coordinate frames and vectors to build up the kinematic model of the length measurement.

This work treats the estimation problem of the position and orientation (pose) of the upper platform of a tendon-driven continuum mechanism. The considered mechanism is depicted, in its current application in Figure 1, and a schematic drawing to illustrate the kinematics in Figure 2. It consists of a inertial fixed lower platform, a moving upper platform and a continuum structure in between. The inertial frame of reference is denoted ℬ and is attached to the base of the neck. The position of the upper platform is described by the origin of frame ℋ, expressed in ℬ, and is denoted as _ℬ_ $r={\left(x,y,z\right)}^{T}$. The orientation of frame ℋ, expressed in ℬ, is represented by the three Euler angles, denoted as $\theta ={\left(\theta_{x},\theta_{y},\theta_{z}\right)}^{T}$, and combined with the transformation order

$$A_{\mathrm{ℬℋ}}=R\left(\theta_{z}\right)R\left(\theta_{y}\right)R\left(\theta_{x}\right)$$ (1)

where $A_{\mathrm{ℬℋ}}\in {\mathbb{R}}^{3\times 3}$ incorporates the base vectors of frame ℋ. As the workspace of the system does not exceed $\pm 90^{\circ }$ in any direction, no singular configurations of the Euler angles appear. The pose is summarized in the vector $q\in {\mathbb{R}}^{6}$,

$$q={\left(x,y,z,\theta_{x},\theta_{y},\theta_{z}\right)}^{T}$$ (2)

For actuation, tendons are connected to the upper platform. By putting tension on them, a loading is introduced onto the upper platform which deforms the continuum and initiates a motion. The tendons are routed alongside the continuum to the lower platform without touching, Figure 1 indicated with red. In the lower platform, the tendons actuators are located. The incorporated position measurement $\Delta \tilde{l}\in {\mathbb{R}}^{4}$ allows the free length of each of the tendon to be measured, assuming a known initial length $l_{t,0}\in {\mathbb{R}}^{4}$,

$$l_{t}=l_{t,0}-\Delta \tilde{l}$$ (3)

Furthermore, four additional length sensors are placed on the system indicated in green in Figure 1. They are also spanned from the upper to the lower platform and provide sensor values $\tilde{s}\in {\mathbb{R}}^{4}$ which correspond linearly to their length,

$$l_{s}=K_{s}\tilde{s}$$ (4)

with the constant calibration matrix $K_{s}\in {\mathbb{R}}^{4\times 4}$. In summary, the sensor information are included in $\tilde{l}={\left(l_{t}^{T},l_{s}^{T}\right)}^{T}\in {\mathbb{R}}^{8}$ and as the attachment and routing points of additional length sensors are known, a geometric model for the measured length is available,

li(q)=‖rp,i−ℬr−Aℬℋℋri‖2,for i=1...8 (5)

In a prior publication (Deutschmann et al., 2019), the estimation problem was solved using the geometric model for the length sensors of Eq. 5. An online minimization of the error $e_{l}\in {\mathbb{R}}^{8}$ between the length measurements $\tilde{l}$ and the model l(q) is carried out to find an estimate of the pose $\hat{q}\in {\mathbb{R}}^{6}$,

$$\underset{\hat{q}}{\min}{\left\|\tilde{l}-l\left(q\right)\right\|}_{2}^{2}=\underset{\hat{q}}{\min}{\left\|e_{l}\right\|}_{2}^{2}$$ (6)

Sources of error using this pose estimation technique are related to the following assumptions. First, it is assumed that the sensor readings in Eqs 3, 4 correspond linearly to a length of the tendons or sensors. Second, the geometric model in Eq. 5 relies on the accurate knowledge about the pulley kinematics of the tendons, and perfectly known attachment points and straight sensors. If these assumptions do not hold, the accuracy of the resulting pose which minimizes Eq. 6 might be affected.

In tests on the real hardware, the pose could be predicted with a maximum estimation error of ±4.5 mm for position and ±4° orientation (Deutschmann et al., 2019). The drawbacks of this method are that a good initial estimate is required to converge, the presence of local minima, and the assumption of perfectly known dimensions, e.g., the location of the pulleys and hinges w.r.t. ℬ.

## 3 Related Work

There are two main approaches to pose estimation for elastic structures. If the geometric and material parameters are known with high accuracy, geometric (Jones and Walker, 2006) or static (Camarillo et al., 2009; Rucker and Webster, 2011) deformation models are employed. As these models require information about actuator positions or forces/torques, a common strategy is to use actuation sensors or additional sensors such as passive cables (Rolf and Steil, 2012) or fiber-bragg sensors (Roesthuis and Misra, 2016). More recently, a pose estimation technique without a deformation model for tendon-driven continuum mechanism was proposed (Deutschmann et al., 2019). It uses encoder values of the tendon actuation, additional deformation based length sensors and a model for the pose-dependent length measurements to extract the pose by nonlinear optimization or an Extended Kalman Filter. Two disadvantages of model-based approaches is that they are prone to parameter uncertainty, and also rely on the assumption of taut tendons (and length sensors) which might be jeopardized in fast motions or external contacts.

The second approach is to use data-driven methods, which learn a direct input-output behavior from measured data. The inputs are commonly actuation torques or actuator positions, and the corresponding output is the end effector pose. Popular models are based on neural networks to approximate the static characteristics from actuation forces (Braganza et al., 2007; Giorelli et al., 2015), or a Gaussian mixture model to relate actuator lengths to end effector poses (Malekzadeh et al., 2016).

Fault detection in robotic systems (Khalastchi and Kalech, 2018) is a particular instance of the wide field of anomaly detection (Chandola et al., 2009). Three main approaches to tackle the issue are distinguished (Khalastchi and Kalech, 2018): 1) knowledge-based, 2) data-driven-based and 3) model-based approaches.

The fundamental assumption in knowledge-based approaches are that all faults and their corresponding symptoms are known. Then, casual analysis is used based on the fault-symptom relationship to find to fault which is occurring. In (Hamilton et al., 2001) this approach is used to for autonomous underwater vehicles.

Similarly, a common technique in machine-learning is train a model to classify normal and abnormal behaviors in a supervised fashion (Hornung et al., 2014). This requires a labeled dataset, and is unlikely to generalize to unseen faults. When no labels are available, unsupervised methods such as distance to a nearest neighbor (He and Wang, 2007) or data clustering (e.g., fitting a Gaussian mixture model to the data (Yu et al., 2010)) can be used instead.

Machine learning techniques belong to the second category, i.e., data-driven approaches and represent a large source for possible fault detection techniques. Other approaches covers the generation of data for nominal behavior by a physical simulator (Haidu et al., 2015). Based on the data, a failure envelope is learned for each datatype and failures are distinguished if the envelope is violated during task execution.

Other data-driven approaches make use of statistical filters. A common approach in mobile systems is the usage of Kalman-filters. A fast approach which utilizes one Kalman filter can be found in (Schmid et al., 2012; Steidle et al., 2016). Faulty sensors used in the process update are handled based on a confidence measure of the sensor data. Depending on that confidence, sensor information is added or removed in the process update. Also, a bank of Kalman-filters is utilized where each Kalman-filter predicts the nominal state of a systems assuming a specific failure has occurred. For a mobile autonomous robot in (Goel et al., 2000), a bank of eight filters are utilized (implying eight possible failure sources) and the most reliable filter, used for state estimation, is chosen based on pre-trained neural-network using the filter residuals. As every filter is related to a specific failure, diagnosis is already incorporated.

The last category are model-based approaches. Commonly, they utilize a model of the system for a nominal behavior and fault are correspondingly identified if the real behavior, measured by some signals, is not coherent with the model of this signal. For hardware failures, physically inspired models are commonly used and a comprehensive treatment can be found in (Chen and Patton, 2012).

Our proposed method is a data-driven approach which utilizes machine learning with unsupervised training as it does not require labels. Unlike the previous approaches, that only detect a potential failure, we leverage minimal knowledge about the task to detect and handle the fault.

## 4 Method

### 4.1 Pose Estimation as a Regression Problem

The problem of predicting the six-DoF pose q given measurements $\tilde{l}$ can be formulated as a supervised learning problem. For a given estimator $f_{\Theta }$, parametrized by the vector $\Theta \in {\mathbb{R}}^{p}$ (p is the number of parameters), the objective is to find the parameters Θ that minimize the error between its prediction $\hat{q}=f_{\Theta }\left(\tilde{l}\right)\in {\mathbb{R}}^{6}$ and the true pose q:

$$ℒ\left(q,\hat{q}\right)=\text{‖}\;q-{\hat{q}\;\text{‖}}_{2}^{2}$$ (7)

where ℒ is the loss function or objective function.

This is a classic regression problem that can be solved using various techniques (Stulp and Sigaud, 2015). In this paper, we use linear models $f_{\Theta }\left(\tilde{l}\right)=\Theta^{T}\tilde{l}$ and second-order polynomial models $f_{\Theta }\left(\tilde{l}\right)=\Theta^{T}\phi \left(\tilde{l}\right)$, where ϕ extracts polynomial features. We estimate the parameters with least squares. We select those models and method because they are fast to compute, do not require hyperparameter tuning or a large amount of data, and the learned model is easily interpretable. In our case, we also found that adding more complexity (e.g., using a neural network or higher order polynomial) did not improve accuracy.

### 4.2 Uncertainty Estimation Using Bootstrapped Ensemble

Querying only one predictor $f_{\Theta }$ may be fastest, but one predictor alone does not provide uncertainty estimation, which is crucial to detect and handle failures. Here, we distinguish between aleatoric and epistemic uncertainty (Gal, 2016). Aleatoric uncertainty is due to the sensor noise and is irreducible (unless we improve the sensor precision). Epistemic uncertainty corresponds to the uncertainty in the model. This can be reduced by providing more training data, and increases with input samples out of the training distribution (Kendall and Gal, 2017). This is the uncertainty that allows failure detection.

Uncertainty estimation can be achieved by training an ensemble of n models $\varepsilon =\left\{f_{\Theta_{1}},\;\;f_{\Theta_{2}},\ldots \;f_{\Theta_{n}}\right\}$, each model having different parameters $\Theta_{i}$ (e. g., by using different parameter initialization for each model). A measure of the uncertainty would be the variance of the predictions. That is to say, the uncertainty for dimension k of the pose $q\in {\mathbb{R}}^{6}$ can be estimated with:

$$\sigma_{k}^{2}=\frac{1}{n}\sum_{i=1}^{n}{\left[f_{\Theta_{i}}{\left(\tilde{l}\right)}_{k}-{\bar{f_{\Theta }\left(\tilde{l}\right)}}_{k}\right]}^{2}$$ (8)

where $\bar{f_{\Theta }\left(\tilde{l}\right)}$ is the mean of the predictions and the subscript k denotes the k-ith element of the vector.

**FIGURE 3:**
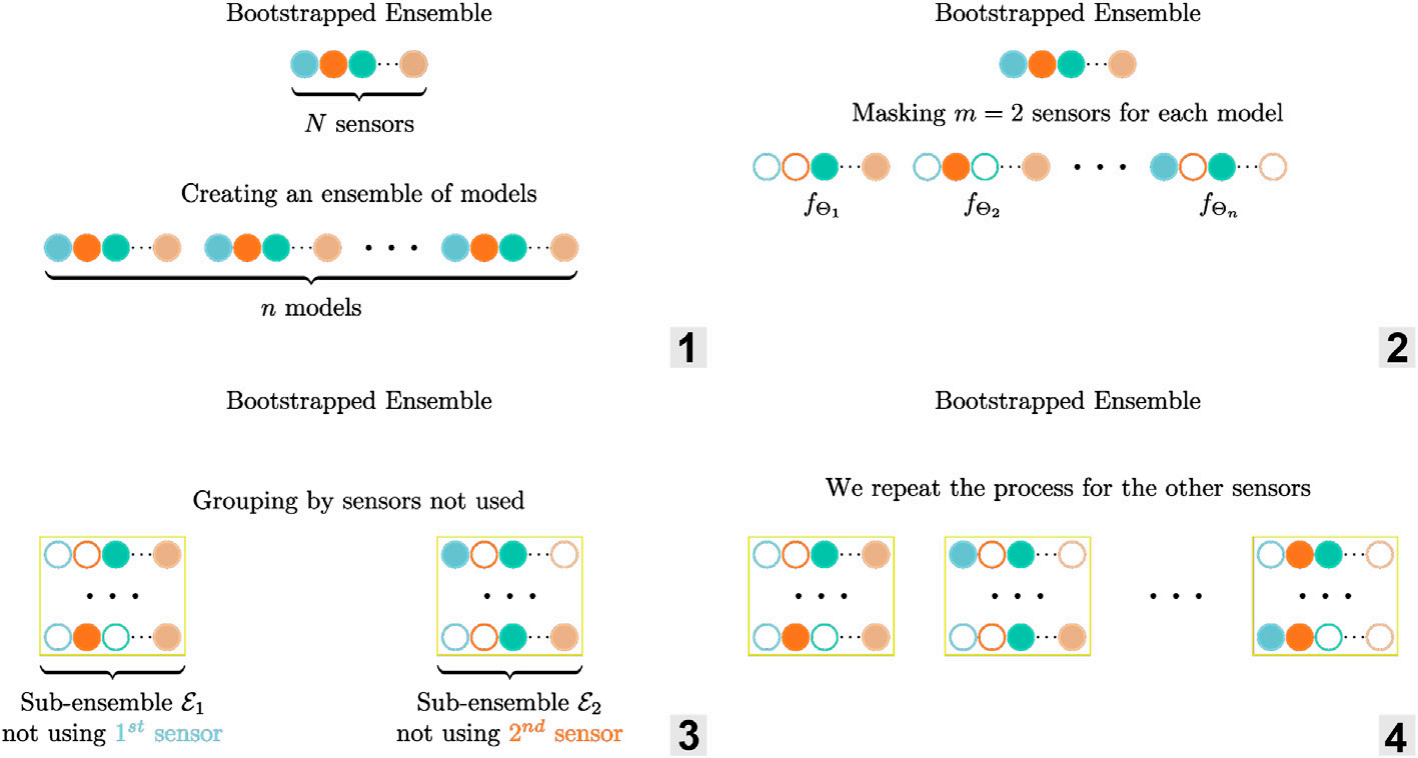
Illustration of the ensemble ε and sub-ensembles creation for m=2 masked sensors per estimator. 1. Creation of an ensemble of models 2. Bootstrapping by masking sensors 3–4. Grouping the ensembles by sensor not used to create sub-ensembles.

Unfortunately, such an ensemble underestimates the epistemic uncertainty, as all the models are trained on the same dataset D. To address this issue, we can train each model $f_{\Theta_{i}}$ only on a subset of the training set $D_{i}\subset D$. That way, each model makes different errors and it reduces the overconfidence of the ensemble. This technique is called bootstrapping (Efron, 1992; Breiman, 1996) and the resulting ensemble is a bootstrapped ensemble. The interesting bit of bootstrapped ensemble is that we can use any predictor $f_{\Theta_{i}}$. Figure 3 summarizes the creation of ensembles and sub-ensembles of models.

### 4.3 Failure Detection and Handling

**FIGURE 4:**
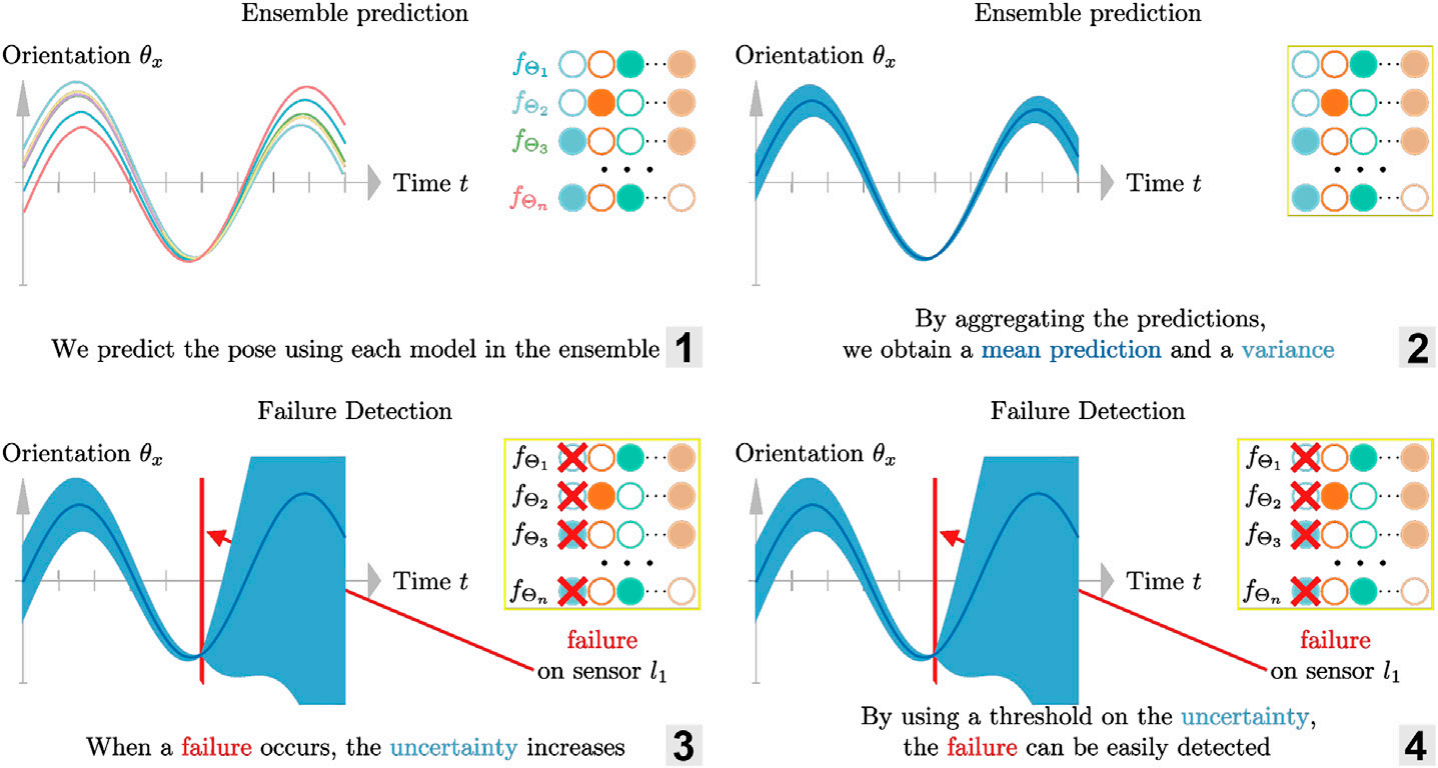
Illustration of the failure detection using an ensemble of estimator. 1. Predicting the pose which each model in the ensemble 2. Aggregating the predictions to obtain a mean and a variance 3–4. Detecting failure by using a threshold on the variance (measure of uncertainty).

The ensemble of estimators presented in the previous section gives us an uncertainty measure: the variance $\sigma_{k}^{2}$ of the estimators. With this uncertainty measure, we can simply use a threshold λ to detect a failure. That is to say, we consider that there is a failure if $\sigma_{k}^{2}>\lambda$. Figure 4 illustrates how such ensemble can be used to detect a failed sensor.

For our six-DoF pose estimation problem, we have redundant sensor information: there are more sensors than needed and there is coupling in the system. Because of the extra sensors, we can detect which one(s) failed, and react to it by grouping the predictions. This allows to estimate the pose q accurately even with multiple failures. The key insight is how we define the subsets $D_{i}\subset D$ to create the ensemble (cf. Figure 3): we use only part of the sensors measurements $\tilde{l}$ to construct the subsets and then group the estimators in sub-ensembles $\varepsilon_{l}\subset \varepsilon$ by sensor(s) not used for prediction. As an example, masking the first sensor results in the input vector $\tilde{l}=\left(0\;\;\tilde{l}{\;}_{2}\;\;{\tilde{l}}_{3}\;\;...\;\;{\tilde{l}}_{8}\right)$.

**FIGURE 5:**
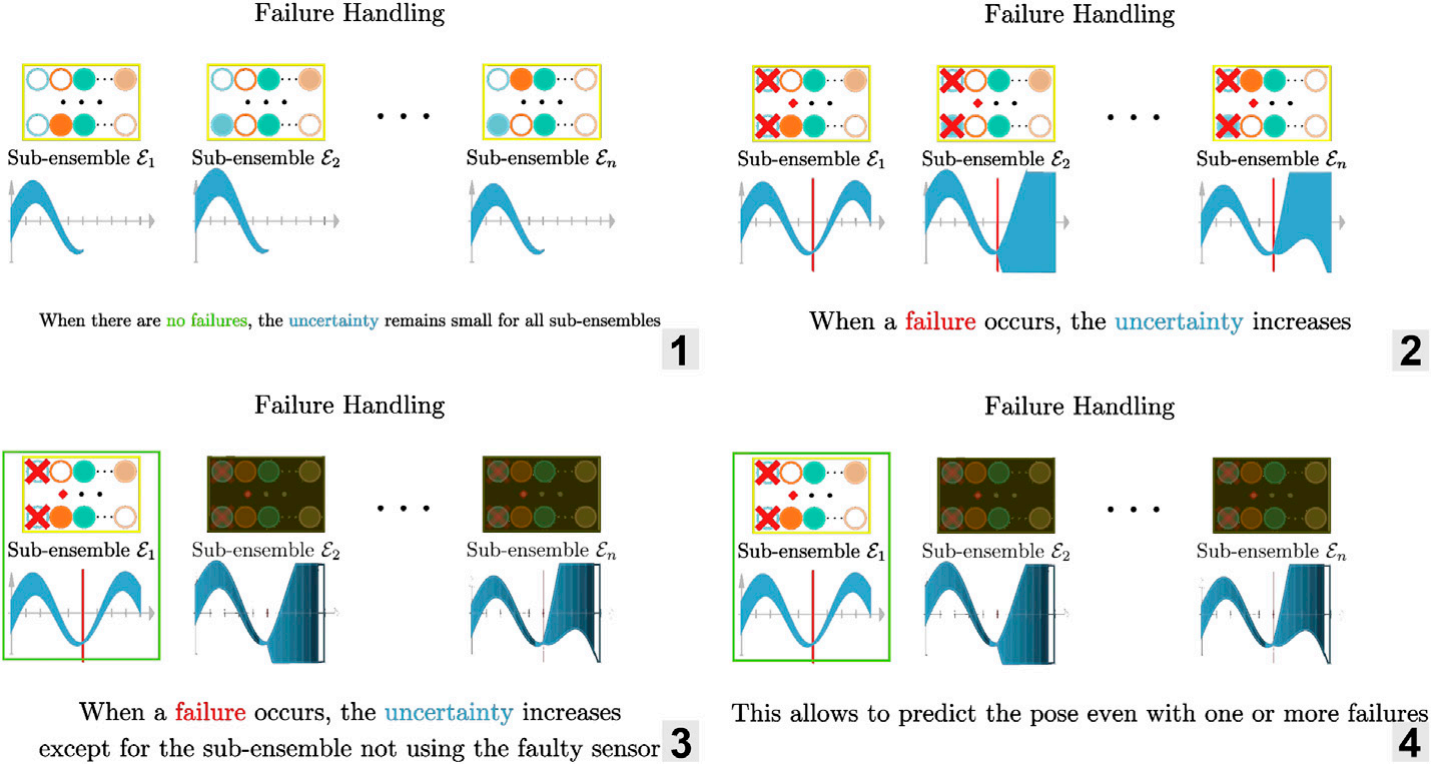
Illustration of the failure handling using an ensemble of estimator. 1. Predicting the pose using each sub-ensembles when there is no failures: the uncertainty remains small 2–3. When a failure occurs, the uncertainty increases except for the sub-ensemble not using the faulty sensor 4. Because there is a sub-ensemble not affected by the failure, we can continue to predict accurately the pose.

To illustrate the idea, we now consider the case where we mask m=2 sensors (out of N=8) as shown in Figures 3, 5. Among the ensemble of trained models $\varepsilon =\left(f_{\Theta_{i}},i=1\ldots n\right)$, one is not using sensors ${\tilde{l}}_{1}$ and ${\tilde{l}}_{2}$ (masked sensors are filled with black in Figure 3), another not using ${\tilde{l}}_{1}$ and ${\tilde{l}}_{3}$, … in a way that there is a sub-ensemble $\varepsilon_{{\tilde{l}}_{1}}\subset \varepsilon$ composed of estimators that don’t use the first sensor ${\tilde{l}}_{1}$ for predicting the pose (first sub-ensemble in Figure 3), a second one $\varepsilon_{{\tilde{l}}_{2}}$ with estimators that don’t use the second sensor ${\tilde{l}}_{2}$, … as a result, if one sensor breaks down as in Figure 5, we can still use the predictions of one sub-ensemble: the one that does not use the failed sensor.

In order to check if a sub-ensemble $\varepsilon_{l}$ is usable, we compute the sub-ensemble variance $\sigma_{k}^{2}$ and compare it to a threshold . When there is no error, all sub-ensembles should have a variance below the threshold. If one failure occurs, e. g. on the sensor j, then only one sub-ensemble should pass the test: $\varepsilon_{{\tilde{l}}_{j}}$ which is composed of estimators that do not use sensor j. The appropriate threshold λ is determined empirically.

The previous example was for one failed sensor only. To detect and handle more than one, we repeat three steps:

1. Ensemble prediction: predict the pose using each model from the ensemble ε
2. Group predictions by sensors not used to create the sub-ensembles $\varepsilon_{l}\subset \varepsilon$
3. Check the variance of each sub-ensemble to detect failure

We start with m=2 masked inputs to detect and handle one failure and increment that number if needed. We provide the reader with a visual explanation of the method in the Supplementary Video.

To detect $n_{failures}$ failures, we need to create sub-ensembles $\varepsilon_{l}$ with $m=n_{failures}$ sensors masked. To identify which sensor(s) failed, we need to mask one additional sensor. As a first approach, we create the subsets $D_{i}\subset D$ corresponding to all the possible $\left(\begin{array}{c} N\\ m \end{array}\right)$ mask configurations, and train one model for each subset. That is to say, we train an ensemble ε of $n=\left(\begin{array}{c} N\\ m \end{array}\right)$ models.

We then repeat that process with additional sensors masked, until there are not enough sensors left to have a reliable prediction. For the experimental platform used in this paper, the experiments reveal that relying on 3 (out of N=8) sensors is enough to have an acceptable precision, which means we can detect and handle up to 4 failures.

Concretely, to be robust up to 4 failures, we need to cover the cases where:

• there is one failure, therefore mask 2 sensors and train $\left(\begin{array}{c} 8\\ 2 \end{array}\right)=28$ polynomial models.
• there are two failures, therefore mask 3 sensors and train $\left(\begin{array}{c} 8\\ 3 \end{array}\right)=56$ polynomial models.
• there are three failures, therefore mask 4 sensors and train $\left(\begin{array}{c} 8\\ 4 \end{array}\right)=70$ polynomial models.
• there are four failures, therefore mask 5 sensors and train $\left(\begin{array}{c} 8\\ 5 \end{array}\right)=56$ polynomial models.

Which means we have to train $\sum_{m=2}^{5}\left(\begin{array}{c} N\\ m \end{array}\right)=210$ polynomial models in total to handle all possible cases. We start with m=2 masked sensors and the first 28 polynomial models (nominal case, no failure) and then increment the number of masked sensors and use additional polynomial models only if needed. The failure detection and handling process remains the exact same at each stage: they are the three steps presented above.

Although simple, this naive way of creating subsets does not scale well if the number of sensors or failures handled increases. We discuss in Section 5.6 how to optimize that process.

## 5 Experiments

The goal of this section is to evaluate the performance of the proposed method in terms of speed and accuracy, and investigate its robustness against one or more sensor failures.

### 5.1 Experimental Setup

The experimental setup consists of the tendon-driven continuum mechanisms used as the neck of the humanoid robot DAVID, Figure 1. The upper platform is equipped with a marker target of an external camera tracking system, which serves as the ground truth data of the pose. The tracking system is only used for training, evaluation and is not needed afterward. The pose dependent length information that is retrieved at a frequency of 300 Hz comes from two sources. The tendon lengths are given by the tendon actuators from Robodrive and the four additional length sensors are provided by Kinfinity UG.

The placement of the two type of sensors is not arbitrary. The tendons, i.e., the power transmission element of the actuation, decide the reachable workspace of the mechanism and therefore their routing cannot be changed. In contrast the placement of length sensors can be chosen almost freely. In (Deutschmann et al., 2019), different placements of the sensors were experimentally investigated regarding their accuracy. To provide a fair comparison, the configuration yielding best results was also chosen for the present paper. The system is driven to different poses or along trajectories by commanding different sets of tendon-tension, which are realized by a local tendon-tension controller in each of the actuators (Chalon et al., 2011).

### 5.2 Data-Driven Pose Estimation

#### 5.2.1 Static and Dynamic Estimation Error

*Static Pose Estimation*. To assess the performance of our six-DoF estimator and compare it to previous work, we first command the neck to reach 200 static poses. For each pose, we retrieve the ground truth q using an external camera tracking system from Nikon (Metris, 2016) and compute the prediction error in position and orientation. To train the models, we sample randomly 20 poses and use the rest of the datapoints for testing. Each model is therefore trained with the same 20 datapoints, but each one with different sensors masked, so each model uses a different set of input features. We repeat that process using 10 different random seeds. To estimate the runtime of each method, we perform 1,000 single predictions and average the time taken. The test is done on a computer equipped with 8 Intel i7-8550U CPUs at 1.80 GHz.

*Dynamic Pose Estimation*. We then evaluate the pose estimation method trained with static poses only on dynamic motions. For that, we record the ground-truth and estimated pose on a pre-defined trajectory. We provide the reader with a Supplementary Video showing the method in action.

#### 5.2.2 Results

**FIGURE 6:**
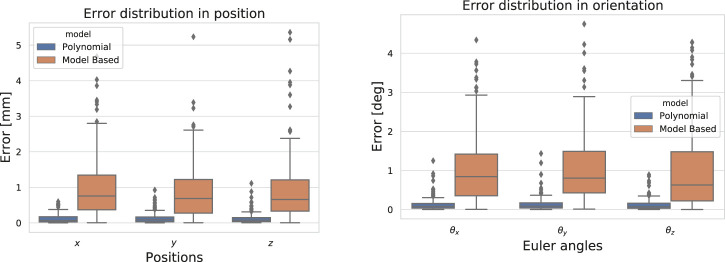
Error distribution in position and in orientation on 180 static poses for the model-based approach (Deutschmann et al., 2019) and the polynomial model.

*Static Pose Estimation*. The results are summarized using mean error and standard deviation over the test poses in Table 1, runtimes are included when available. We also show the error distribution in position and orientation in Figure 6.

**TABLE 1:** Comparison of mean runtime and error (both in position and orientation) for each method. The data-driven approaches are fast and also more accurate than the model-based approach. For each metric, we bolded the best mean. “N/A” means that the data is not available.

	Model-Based Deutschmann et al. 2019	Linear	Polynomial
Runtime (ms)	N/A ± N/A	**0.1 ± 0.0**	0.2 ± 0.0
Static Pose Estimation
Error in position (mm)	1.1 ± 1.0	0.3 ± 0.3	**0.2 ± 0.2**
Error in orientation (deg)	0.9 ± 0.8	0.3 ± 0.3	0.1 ± 0.1
Dynamic Pose Estimation			
Error in position (mm)	5.1 ± 2.3	1.7 ± 1.4	**1.6 ± 1.4**
Error in orientation (deg)	3.8 ± 3.0	0.8 ± 0.6	**0.5 ± 0.4**

Overall, the data-driven approaches are fast (they run at ∼ 5000 Hz) and more accurate than the model-based one: the mean error is reduced up to 5 times (cf. Table 2). As expected, the linear model runs faster at a cost of some accuracy compared to the polynomial model.It is worth mentioning that although the model-based approach appears inaccurate in the present comparison, it is fairly accurate and fast to compute when compared to other model-based pose estimation techniques as reported in (Deutschmann et al., 2019).

**TABLE 2:** Ablation study: influence of the amount of training data, number of sensors and type of model on the performance. We bolded results with the best mean error. Baseline models are trained using 20 datapoints and 8 sensors.

	Position error (mm)	Orientation error (deg)
Model-Based	1.1 ± 1.0	0.9 ± 0.8
Linear baseline	0.3 ± 0.3	0.3 ± 0.3
Polynomial baseline	0.2 ± 0.2	**0.1 ± 0.1**
Neural network (2 layers)	0.8 ± 0.6	0.8 ± 0.7
Linear (3 sensors)	1.0 ± 0.7	1.2 ± 1.0
Linear (4 sensors)	0.4 ± 0.3	0.5 ± 0.6
Polynomial (3 sensors)	0.9 ± 0.6	1.2 ± 0.9
Polynomial (4 sensors)	**0.1 ± 0.1**	**0.1 ± 0.1**
Polynomial (5 datapoints)	0.9 ± 0.9	1.1 ± 0.9
Polynomial (10 datapoints)	0.5 ± 0.5	0.6 ± 0.5
Polynomial (40 datapoints)	0.2 ± 0.3	0.3 ± 0.2

**FIGURE 7:**
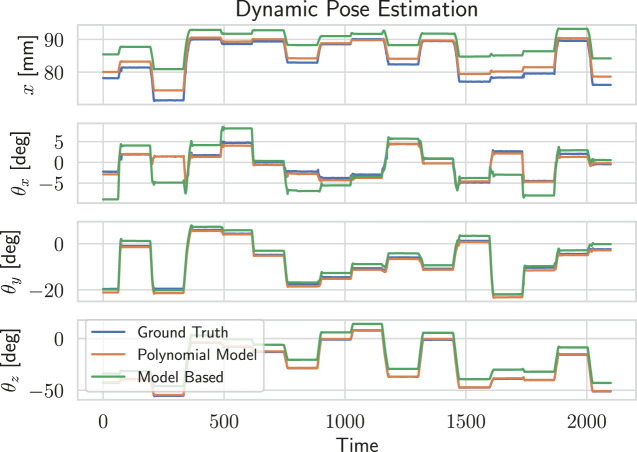
Qualitative comparison of the model-based and polynomial estimators for dynamic pose estimation.

*Dynamic Pose Estimation*. To report also on the dynamic estimation behavior, the experimental platform is driven to several static poses subsequently and the estimated pose for the model-based and the present approach are recorded. The mean-error along the trajectory of 200 subsequent poses is given in Table 1 and the corresponding trajectories of four estimated coordinates $\left(x,\theta_{x},\theta_{y},\theta_{z}\right)$ can be seen in Figure 7.

As for static poses, the data-driven approaches perform best, with the polynomial model being more accurate than the linear one. Mean error reported in Table 1 is higher than for static poses for two reasons. First, it now accounts for the transition error between two static poses, that are not covered at all during training. Then, as we are considering trajectories, static errors at fixed poses accumulate yielding a higher mean value.It is worth noting that those models were only trained on static poses and therefore the results could be improved if part of the dynamic poses where included in the training set.

### 5.3 Hyperparameters Study

To study the effect of the different hyperparameters (type of model, training set size, number of sensors) on the performance, we compare the baseline regressors (20 datapoints and 8 sensors) to several variants. One datapoint corresponds to training data at a specific pose. We present the results in Table 2.

*Effect of the training set size*. We vary the training set size from 5 datapoints to 40 datapoints for the polynomial model. Overall, with more training data, the performance improves. However, after a certain amount (here 30 datapoints), adding more data does not improve the results anymore. This is due both to the irreducible error (sensor noise) and to the limited capacity of the polynomial model. Although the results are slightly worse with 40 datapoints, the difference is not significant.

*Effect of the type of model*. We compare linear, polynomial and neural network^1^ models. Although the neural network yields good performance, it is less sample efficient than the two others, i. e., with more training data it would reach the same accuracy. It also requires hyperparameter tuning (learning rate, mini-batch size, …) and has more parameters (∼3e6 parameters vs. 276 for the polynomial model). For those reasons, using a polynomial model of order 2 is enough in our case.

*Effect of the number of sensors*. We compare the baseline linear and polynomial models (8 sensors) to models using less sensors (3 and 4 sensors). As expected, adding more sensors reduces the error for the linear model. Almost no changes can be observed for the polynomial model with more than 4 sensors: the results are slightly worse but the difference is not significant. As discussed in Section 4.3, having redundant sensor information is key to detect and handle potential failures. The more sensors we have, the more failure we can handle. Therefore having 8 sensors is preferable.

*Effect of the placement of sensors*. Because the polynomial model performs well with only 4 sensors (for instance when using only the tendon length sensors), the placement of the 4 additional length sensors will not affect much the accuracy. However, the positioning would affect the ability to detect failures: if the information that a sensor provides is not useful for the prediction, then it will not be used by the polynomial model, the weight for this input feature will be close to zero. As a result, if such sensor fails, as it does not affect the variance of the predictions, the failure will not be detected but the pose will still be predicted accurately.

### 5.4 Failure Detection and Handling

**FIGURE 8:**
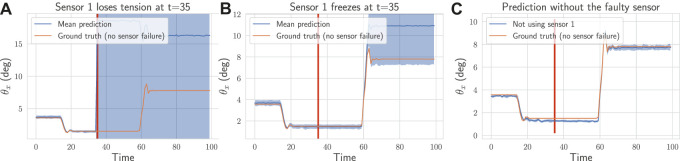
On a recorded trajectory, we simulate two types of failures (depicted as the red vertical line): **(A)** one tendon goes slack at t=35 and the associated sensor ${\tilde{l}}_{1}$ outputs zeros **(B)** the sensor ${\tilde{l}}_{1}$ freezes at t=35 and outputs a constant value. In **(A)** and **(B)**, we plot the mean prediction (dark blue) from the ensemble of estimators along with the uncertainty (shaded blue area). In both cases, the uncertainty goes above the threshold and the method is able to detect the failures and select the sub-ensemble not using the faulty sensor. In **(C)**, we plot the prediction of that sub-ensemble along with the corresponding ground truth. The method is able to predict accurately the pose even with a broken sensor.

**FIGURE 9:**
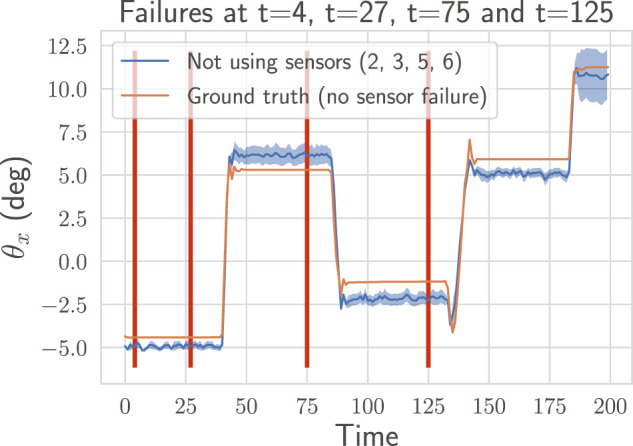
On a recorded trajectory, we simulate four failures (depicted as the red vertical lines). The proposed method detects automatically those four failures (sensors ${\tilde{l}}_{6}$ at t=4, ${\tilde{l}}_{2}$ at t=27, ${\tilde{l}}_{5}$ at t=75 and ${\tilde{l}}_{3}$ at t=125), selects the sub-ensemble not using the faulty sensors and continue to predict accurately the pose. We plot the pose prediction with mean and variance for the sub-ensemble of estimators selected by our method.

To evaluate the effectiveness of the proposed approach for detecting and handling failures, we simulate the loss of sensors while the neck is moving. A common failure is when a length sensor outputs wrong values because a tendon goes slack. Instead of a correct measurement, it outputs zeros. Another type of failure, harder to detect, is when the sensor freezes and outputs a constant value. In Figures 8A,B, we show the effect of both failures on the uncertainty: a jump can be observed right after the loss of tension in Figure 8A. When the sensor freezes (cf. Figure 8B), as expected, the failure is detected only when the neck position changes. Because in both cases the variance increases by a large amount, no careful tuning of the detection threshold is required. To show that the method can handle more than one failure, we simulate in Figure 9 the loss of four sensors.

In Figures 8C, 9, we display the prediction over time for the sub-ensemble not using the broken sensors. We use the ground-truth pose as a reference. The approach successfully detect all the failures and handle them by using the sub-ensemble not affected by the loss of the sensors. As a result, the proposed method can robustly and still accurately (cf. Table 2) predict the pose even with multiple losses.

### 5.5 Comparison With the Linearized Model

The geometry of the deformation of the neck system and the coupling of the tendon motion is highly nonlinear (Deutschmann et al., 2018). The geometrical exact nonlinear mapping can be found in (Deutschmann et al., 2019). This work confirms the non-linearity as a polynomial model of order two reveals the best prediction results. However, as stated in Table 2, the linear models already yield results comparable to the model-based approach. We therefore compare the first-order term of the trained linear coefficients for four sensors used with the partial derivative of the q(l) i. e., a local linearization about the initial straight configuration in the following equation.

$$f_{lin}=\left(\begin{array}{cccc} 0.41 & 0.35 & 0.37 & 0.41\\ -0.25 & 0.30 & 0.25 & -0.26\\ 0.38 & 0.56 & -0.56 & -0.38\\ 7.46 & -16.61 & 15.76 & -7.51\\ -9.94 & -5.17 & 5.05 & 9.63\\ -4.13 & 7.75 & 5.45 & -3.45 \end{array}\right)$$ (9)

$${\left(\frac{\partial q\left(l\right)}{\partial l}\right)}^{T}=\left(\begin{array}{cccc} 0.44 & 0.06 & 0.06 & 0.44\\ -2.33 & 2.5 & 2.50 & -2.34\\ 0.01 & 1.78 & -1.73 & -0.01\\ 0.43 & -0.15 & 0.15 & -0.43\\ -11.79 & -1.0 & 0.99 & 11.76\\ -2.69 & 2.78 & 2.77 & -2.63 \end{array}\right)$$ (10)

The top three rows correspond to positions and the bottom three rows to orientations. The trained coefficients (9) and the model-based coefficients (10) have the same signs and symmetries, indicating that the sensor information is used in a similar fashion to predict the direction of the pose. However, the magnitude of the values defers largely: simply linearizing the model would not be as accurate as the trained linear model.

### 5.6 Limitations

We have shown in the previous sections that the proposed approach yields a good estimation of the pose and handle failures while keeping a low runtime. However, we have to train $\sum_{m=2}^{5}\left(\begin{array}{c} N\\ m \end{array}\right)=210$ models to handle all possible failures. We can optimize our approach to make it scalable both in term of number of sensors and failures handled. A first optimization is to restrict the sub-ensembles $\varepsilon_{l}$ to a maximum of two models. Then, to handle more sensors, we can cluster them (e.g., grouping them in a hierarchical way) or compress the information to reduce the input dimension (e.g., using an auto-encoder). Doing so, our approach would identify a failure from a group of sensors and no more from a single sensor.

## 6 Discussion and Conclusion

In this work, we show that data-driven approaches are competitive alternatives to estimate the pose of a tendon-driven continuum mechanism. The linear and polynomial models are fast to train, require only a small amount of data and prove to be more accurate that the model-based approach. As mentioned in the end of Section 2, assumptions about the sensor linearity and perfectly known kinematics are made which, in cases where the loose their validity, may result in larger errors in the pose estimation. In other words, small deviations or modeling errors in the kinematics of the tendon pulleys or the attachment points of the sensors will cost accuracy in the estimated pose. In contrast, the polynomial model learns a direct mapping from sensor readings to the measured pose and does not make these assumptions, allowing an improved accuracy.

To detect and handle sensor failures, we make use of ensembling technique and minimal knowledge about the system. By clustering the different models, our method predicts the pose accurately even with 4 out of 8 faulty sensors.

The presented method embodies a computationally fast and accurate pose estimation method. This method was already employed to train a reinforcement learning controller (Raffin and Stulp, 2020) and could also replace the model-based approach, currently used in the model-based control approach implemented on the neck system (Deutschmann et al., 2017). This would result in a more accurate positioning given the more accurate estimated pose.

One limitation is the scalability of the method, as discussed in the previous section, which should be addressed in the future. Apart from that, our method is in fact not specific to tendon-driven continuum mechanism: it only requires sensor redundancy and learn to predict the pose directly from data.

## Data Availability

The raw data supporting the conclusions of this article will be made available by the authors, without undue reservation.

## Footnotes

1 2 fully-connected layers of 256 units each, trained with Adam optimizer until convergence using a learning rate of 1e−3 and a batch size of 20 (size of the training set). Different hyperparameters where tested, and these performed best.
